# Editorial: Polymer Surface Chemistry: Biomolecular Engineering and Biointerfaces

**DOI:** 10.3389/fchem.2019.00271

**Published:** 2019-04-25

**Authors:** Hsien-Yeh Chen, Tomohiro Hayashi, Meike Koenig, James J. Lai

**Affiliations:** ^1^Department of Chemical Engineering, National Taiwan University, Taipei, Taiwan; ^2^Tokyo Institute of Technology, Tokyo, Japan; ^3^Department of Advanced Polymers and Biomaterials, Institute of Functional Interfaces, Karlsruhe Institute of Technology, Karlsruhe, Germany; ^4^Department of Bioengineering, University of Washington, Seattle, WA, United States

**Keywords:** polymer chemistry, biointerface, biomolecular, biomaterials, surface modification

Polymer materials exhibit controllable surface properties and can be used to address sophisticated biointerface problems. Polymers can be exploited in applications requiring malleable properties in both a physically and chemically defined fashion for stem cell niches, e.g., manipulating stem differentiation patterns and/or proliferation activities. In addition, these material properties can be matched to each specific case of a drug/target combination and the desired administration conditions, and the possibility of tailoring polymer materials by varying the chain length, crosslinking density, and/or copolymerization is of uttermost importance in the field. The current research topic of Polymer Surface Chemistry: Biomolecular Engineering and Biointerfaces presents promising and exciting results in the use of polymers and their engineered properties. Wang et al. report on the development of a micellar system that combines a copolymer with a pH-responsive lower critical solution (LCST) temperature [poly-(N-isopropylacrylamide-co-N,N-dimethylacrylamide-co-10 undecanoic acid)] with magnetic carboxymethyl-dextran/Fe_3_O_4_-nanoparticles. The copolymer ratios are optimized for the desired LCST behavior so that the system can be used for the controlled delivery of an anti-inflammatory drug selectively to inflamed tissue, which possesses a higher temperature and lower pH value than healthy tissue. Woo Jung et al. describe the synthesis of bicompartmental fibers by electrohydrodynamic co-jetting with side-by-side needle geometry. These fibers comprise a thermoresponsive poly-(N-isopropylacrylamide)-containing part and a poly(ethylene glycol)-containing part for decoupled drug release. Two different model drugs were loaded onto the bicompartmental nanofiber, and the temperature- and molecular weight-dependent release rates were investigated. In addition, Woo Jung et al. used a multicomponent polymer nanofiber composed of poly(N-isopropylacrylamide-co-allylamine hydrochloride) and poly(ethylene glycol) dimethacrylates. The nanofiber was fabricated via an electrified jetting approach to precisely and simultaneously adjust the composite ratio, fiber geometry, localization, and drug loading. Their results demonstrated a thermally induced actuation mechanism through collapse of poly(N-isopropylacrylamide-co-allylamine hydrochloride) and consequent triggering of drug release. Multiple pharmacokinetics were also enabled by this temperature-dependent trigger and a molecular weight-controlled mechanism to release bovine serum albumin and dexamethasone phosphate.

In the article “*in-situ*-Investigation of Enzyme Immobilization on Polymer Brushes,” Koenig et al. report the immobilization of enzyme molecules onto the surfaces of a polymer brush via covalent bonds without losing enzyme function. Their technique endows polymer surfaces with biological functions, which will aid the development of new biodevices beyond conventional biosensors. Ueda et al. investigate nanosized phase-separated structures of blood-compatible poly(2-methoxyethyl acrylate) (PMEA) in the article "Analysis of Interaction Between Interfacial Structure and Fibrinogen at Blood-Compatible Polymer/Water Interface.” No one had previously expected phase separation of a homopolymer at the polymer-water interface! Moreover, the authors clarify the difference in the interactions of the domains and protein. Their findings may lead to an understanding of molecular processes at the interface of biocompatible polymers with proteins and cells.

In their work on constructing nanostructured bifunctional hydrogels, Kratzer et al. reveal a modification of polyethylene glycol-based hydrogel to include gold nanostructures and chemical immobilization of arginyl-glycyl-aspartic acid (RGD) and Notch ligand Delta-like 1 (DLL1). They report that the polymer and its properties promote the differentiation of hematopoietic stem cells (HSCs) to T cells in ~27 days. Controllable parameters including the immobilized ligand density and the aspect ratio of the gold nanostructure are shown to influence the HSCs. Mantz et al. provided a manuscript on polymer surface modification for substrate-mediated gene delivery. Using RGD-functionalized poly(acrylic acid) brushes they immobilized polyethyleneimine- DNA plasmid complexes on titanium substrates. They found the transfection of NIH/3T3 cells cultured on these surfaces two times higher than on control surfaces.

With respect to the research topic, thriving discussions and research developments are provided, including topological and geometric control from bottom to top, dynamic control of the surface properties toward external stimuli, controlled anisotropy of surface properties (e.g. gradient compositions), multifunctional properties and the control of such properties in multiscale. New ideas and concepts of existing polymer systems and modification techniques are expected to provide complementary performance depending on the encountered biological environment as well as a combinatorial approach for the discovery of prospective polymers and engineering methods.

## Author Contributions

All authors listed have made a substantial, direct and intellectual contribution to the work, and approved it for publication.

### Conflict of Interest Statement

The authors declare that the research was conducted in the absence of any commercial or financial relationships that could be construed as a potential conflict of interest.

